# A potential alternative to traditional antibiotics in aquaculture: Yeast glycoprotein exhibits antimicrobial effect in vivo and in vitro on *Aeromonas caviae* isolated from *Carassius auratus gibelio*

**DOI:** 10.1002/vms3.253

**Published:** 2020-03-24

**Authors:** Ronghua Wu, Junyu Shen, Dandan Tian, Jiaqian Yu, Tao He, Jianhua Yi, Yun Li

**Affiliations:** ^1^ Key Laboratory of Freshwater Fish Reproduction and Development Ministry of Education Southwest University Chongqing China; ^2^ College of Animal Science and Technology Institute of Three Gorges Ecological Fisheries of Chongqing Southwest University Chongqing China; ^3^ Angel Yeast Co., Ltd Yichang China

**Keywords:** *Aeromonas caviae*, antimicrobial drugs, *Carassius auratus gibelio*, yeast glycoprotein

## Abstract

In aquaculture, antibiotics are commonly used to provide protection against pathogens; however, this practice has become controversial due to increased occurrences of microbial resistance, and alternatives are needed. This study aimed to investigate the antimicrobial activity of yeast glycoprotein (YG) against *Aeromonas caviae*. Pathogens were isolated from liver of diseased *Carassius auratus gibelio*. Based on morphological and biochemical analysis, together with 16S rRNA gene sequencing, the isolated strains were identified as *A. caviae* and concluded as clones of a single strain and named L2. Further pathogenicity analysis revealed that *A. caviae* possessed β‐haemolysis, and its median lethal dose for *C. gibelio* was 1.33 × 10^6^ CFU/ml. Hepatic adenylate kinase and pyruvate kinase activities of *C. gibelio* were inhibited post–*A. caviae* infection. Antimicrobial drug test suggested that *A. caviae* was a multidrug‐resistant organism but could be inhibited by YG in vitro. Minimum inhibitory and bactericidal concentration of YG was 83.3 mg/ml and 166.7 mg/ml, respectively. Microbiota sequencing results showed that YG supplement could obviously decrease the relative abundance of *Aeromonas* and increase the microbial diversity. Our study revealed that *A. caviae* from *C. gibelio* was a multidrug‐resistant bacteria strain, and could be significantly inhibited by YG in vivo and in vitro, thus providing important insights into ecological control and pathogenesis of *A. caviae* in aquaculture.

## Graphical abstract

This is the first report that *C. gibelio* is susceptible to *Aeromonas caviae* infection. yeast glycoprotein (YG) supplement could increase microbial diversity and decrease abundance of Aeromonas. *Aeromonas caviae* was multi‐antibiotic and could be inhibited by YG in vivo.

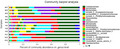

## 1 INTRODUCTION

In past decades, antibiotics have been used in animal feed to reduce bacterial infections. However, this relative practice has been banned in the European Union and United States of America (Pradella, 2006), and become increasingly controversial in the world considering the possible adverse effects of antibiotics, such as developing of resistance strains, contaminating the environment, posing threat to food safety and predisposing to later intestinal disease for reduction in microbial diversity (Cotten et al., 2009; Phillips et al., 2004; Young & Schmidt, 2004). China Ministry of Agriculture has gradually reduced types and doses of antibiotics allowed in feed since 2017 with the intent to ban the usage of antibiotics as animal feed additives by 2020. Thus, finding safe and effective alternatives to traditional antibiotics in aquaculture are needed. Although a lot of attempts have been made over the years, such as using vaccine, probiotics and immunostimulants (Munir, Hashim, Nor, & Marsh, 2018; Wang et al., 2019), the effective alternatives to antibiotics in aquaculture are still to be developed. Yeast glycoprotein (YG) containing glycoprotein and cell wall polysaccharides may increase resistance to infections by Gram‐negative bacteria, and serve as an immunomodulator because the internal β‐dextran can modify immunosuppression (Amorim et al., 2012; Qin et al., 2019). The antimicrobial mechanisms might involve two aspects: (a) glycoprotein could highly gather on the surface of bacterial cell wall by binding to glycoprotein receptor, then interfere with the normal metabolism of bacteria (Amorim et al., 2012); and (b) cell wall polysaccharides activate some cell wall–degrading enzymes, including chitinase and glucanase, resulting the degradation of bacterial cell wall (Fisher, Meroueh, & Mobashery, 2005). Therefore, it might be explored as a natural alternative to prophylactic antibiotics. Besides the role in the digestion and absorption of nutrients, the microbiota in the gut play a critical role in prevention of pathogen colonization. Therefore, research concerning the effect of YG on microbial community will facilitate to illustrate its antimicrobial role. A basal diet containing 800 mg/kg YG has proven effective in improving the gut microbiota in weaned piglets (Qin et al., 2019). However, it is not clear whether YG can play an important role in modifying intestinal microbiota of aquatic animals.

*Aeromonas caviae*, a mesophilic species belonging to genus *Aeromonas*, is one of the most prominent pathogens occurring ubiquitously in the aquaculture industry (Di Ianni et al., 2015; Yi et al., 2013). The diseases caused by *A. caviae* in fish are characterized by bacterial septicaemia or surface ulcer with high mortality (Thomas et al., 2013). So far, great advances have been made with respect to the pathology, epidemiology and diagnostic methods of *A. caviae* (Baldissera et al., 2018; Janda & Abbott, 2010; Schmidt, Bruun, Dalsgaard, & Larsen, 2001), the liver is one of the organs most affected by *A. caviae* (Igbinosa, Igumbor, Aghdasi, Tom, & Okoh, 2012). However, the ecological control and pathogenesis of *A. caviae* in aquaculture are largely unknown.

In this study, YG (Product number: 82001092) was supplied by Angel Yeast Co., Ltd, it mainly included mannan oligosaccharide (≥12%), β‐dextran (≥12%), H2O (≤6%) and crude protein (≤35%), etc. The detailed information of YG was shown in Figure S1. Based on the assumption that the synergistic effect of YG might strengthen the immune function and intestinal microbiota, we hypothesized that YG may be a suitable alternative to antibiotics and reach the similar effects without negative impact when added in the basal diet of aquatic animals. Different from *Aeromonas hydrophila* and *Aeromonas veronii*, research on the pathogenesis, prevention and control of *A. caviae* in fish is limited. To test the in vivo antimicrobial effect of YG, intestine microbial community of *Carassius auratus gibelio* feeding with a basal diet containing antibiotics and YG was determined by 16S rRNA sequencing. Thus, the objective of this study was to evaluate the antibacterial effects of YG in vivo and in vitro on *A. caviae* isolated from *C. gibelio*, the results will provide important insights into disease control and pathogenesis of *A. caviae* in aquaculture.

## 2 MATERIALS AND METHODS

### 2.1 Fish handling and diet

*Carassius auratus gibelio*, an important aquatic species widely cultured in China, was chosen to test the pathogenicity of the aetiological agent isolated from naturally infected *C. gibelio* and in vivo antimicrobial effect of YG. A total of 150 healthy *C. gibelio* with average weight 50 ± 5 g were purchased from a farm in Beibei District of Chongqing. Fish were allowed to acclimate to laboratory conditions for about 2 weeks before artificial infection. Fish were kept in rearing tanks supplied with running water at 25 ± 2°C and fed daily with basal diet (Xu, Wang, Li, & Lin, 2009). The temperature was kept constant for the experimental period.

### 2.2 Aetiological isolation and identification

A total of three naturally infected *C. gibelio* characterized by bacterial septicaemia were obtained from a *C. gibelio* breeding farm in Beibei District of Chongqing municipality, China. Pathogenic bacteria were isolated from liver and inoculated on lysogeny broth (LB) agar medium at 28°C for 24 hr. The typical colonies from three individuals were sub‐cultured thrice to obtain dominant single colonies. A total of three bacterial strains were obtained and named strain L1 to strain L3.

The three bacterial colonies were subjected to Gram stain, biochemical test and 16S rRNA sequencing for identification. Biochemical identification was carried out according to protocols in Bergey's Manual of Determinative Bacteriology (Buchanan & Gibbons, 1974). The purified bacteria and *A. caviae* standard strain were inoculated on indole, VP (voges proskauer), MR (methyl red) and other 29 biochemical identification reaction tubes at 28°C for 12–48 hr.

For molecular identification, bacterial genomic DNA was extracted for 16S ribosomal RNA (16S rRNA) gene amplification following the standard protocol of extraction kit (TIANGEN). The universal primers of 16S rRNA (forward, AGAGTTTGATCCTGGCTCAG; and reverse, ACGGCTACCTTGTTACGACTT) were synthesized by Genomics Institute, Beijing, China. The PCR reaction (50 μl) contained 2 × Taq MasterMix (25 μl), DNA template (100 ng/μl, 3 μl), primers (10 mmol/L, 1 μl) and 20 μl of sterile water. After the initial denaturation (95°C for 5 min), all reactions went through 30 cycles of 94°C for 1 min, 55°C for 1 min and 72°C for 1 min, then 74°C for 10 min. After confirmed by 1% agarose gel electrophoresis, the PCR products were purified using QIAquick PCR purification kit (Qiagen) following the manufacturer's instructions, followed by sequencing in Beijing Genomics Institute, China. The result was compared with the corresponding sequences in NCBI GenBank for homology analysis, and phylogenetic tree was generated by neighbour‐joining method (Saitou & Nei, 1987). Finally, to make it available to other researchers for reproducing our study, *A. caviae* was submitted to China General Microbiological Culture Collection Center (CGMCC) for preservation, which will take about 3 months to give a CGMCC number specific for *A. caviae*. Before the CGMCC number for *A. caviae* is given, other researchers could also get the isolated *A. caviae* strain by contacting the author via email.

### 2.3 Pathogenicity analysis

In vivo, to confirm the pathogenicity of aetiological agent, reproduction of infection was conducted in *C. gibelio* by intramuscularly injecting with serially diluted *A. caviae* (strain L2). Aetiological agent was re‐isolated and identified according to the above‐mentioned methods, and mortality was recorded for calculating median lethal dose (LD50). Briefly, rapidly growing strain L2 was collected from culture medium and enriched by centrifugation at 500 g for 10 min at 4°C, then suspended in 0.01‐M phosphate buffered saline (PBS, pH 7.4). For five experimental groups, fish were injected intramuscularly with 500 μl of serially diluted bacteria suspension (1 × 10^5^, 1 × 10^6^, 1 × 10^7^, 1 × 10^8^ and 1 × 10^9^ CFU/ml), respectively. As the negative control group, fish were injected intramuscularly with equal volume of sterile PBS. Signs were observed twice a day and mortality was recorded for 2 weeks, then LD50 was calculated based on Reed method (Reed & Muench, 1938).

The extracellular products (ECPs) of strain L2 were prepared according to the methods described by Zhang, Pridgeon, and Klesius (2014) with minor modifications. Briefly, strain L2 was inoculated in liquid medium, tryptic soy broth and shake incubated at 28°C. The bacterial suspension was centrifugated at 8,049 g for 30 min and the supernatant recovered was filtered through a 0.22‐μm PES membrane (Millipore), which was referred to ECP. Haemolytic tests of ECPs were carried out in accordance with protocol described in Wang, Xu, Cao, Wang, and Wang (2013). Haemolytic test was conducted in 10% rabbit blood agar medium. The medium plates were inoculated at 28°C for about 36 hr. The occurrence of transparent circle in rabbit blood agar medium indicated positive results of haemolytic activity.

Hepatic adenylate kinase (AK), pyruvate kinase (PK) and adenosine triphosphate (ATP) activities in both infected and uninfected liver of *C. gibelio* were measured according to the published methods (Dzeja, Vitkevicius, Redfield, Burnettm, & Terzic, 1999; Leong, Lai, Lim, & Clark, 1981; Wen et al., 2015). According to the methods, the results of AK and PK activities were expressed in pmol ATP formed/min/mg of protein and μmol pyruvate formed/min/mg of protein, respectively. For ATP levels, the results were reported as pmol/mg of protein.

### 2.4 Antimicrobial activity of antibiotics and YG in vitro

Antibiotic sensitivity was tested by Disc Diffusion Method (Bakht, Islam, Ali, Tayyab, & Shafi, 2011). Pathogenic strains (100 μl, 1 × 10^7^ CFU/ml) were inoculated by spreading method (Elguindi et al., 2011), and the test paper of drug sensitivity was pasted on the LB culture medium at 28°C for 24 hr. According to the America Committee for Clinical & Laboratory Standards Institute (CLSI 2012 M02‐A11 Disc Diffusion Method), the diameter of inhibited colonies circle indicates the antibacterial drug sensitivity.

The antibacterial activity of YG was evaluated according to the Oxford cup method (Wang, Zheng, Li, Wu, & Xiao, 2017). Briefly, the culture dish, Oxford cups (stainless tube with 6 mm in inner diameter, 8 mm in outer diameter, and 10 mm in height), LB medium and other reagents were sterilized in an autoclave before use. The bacterial suspension was adjusted to 10^6^ CFU/ml and coated evenly on culture dish with LB agar medium, then the Oxford cups were placed on the surfaces of the medium and sample solutions of YG (2,000 mg/ml, diluted in sterile distilled water [DW]) were added by dripping. The negative controls were added equal volume of sterile DW. Finally, the culture dishes with the Oxford cups were incubated at 28 ℃ for 24 hr, and the diameter of the bacteria inhibiting loop was measured to evaluate the antibacterial activity of samples. The procedure was performed in triplicate.

The MIC of YG was determined by broth dilution methods according to CLSI (Cockerill et al., 2012). The stock solutions (2,000 mg/ml) of YG were twofold serially diluted in DW to obtain the nine following concentrations: 1,000, 500, 250, 125, 62.5, 31.3, 15.6, 7.8 and 3.9 mg/ml. Nine sterilized glass tubes were added 2 ml of rapid growing *A. caviae* (1.5 × 10^6^ CFU/ml) in LB medium, followed by adding 1 ml of the various dilutions of YG making the final concentrations 333.3, 166.7, 83.3, 41.7, 20.8, 10.4, 5.2, 2.6 and 1.3 mg/ml, and the final concentration of *A. caviae* 1.0 × 10^6^ CFU/ml. For the positive control, additional sterilized glass tube was added 3 ml of 1.0 × 10^6^ CFU/ml *A. caviae*. All the tubes were subjected to shake‐tube inoculation at 28°C for 24 hr, after which all tubes were observed for growth. MBC of YG was derived by sub‐culturing the mixture in LB agar medium at 28°C for 24 hr from tubes that showed no growth (determined by turbidimetric method) in the test of MIC, The minimum concentration of YG that produces total cell death (determined by plate culture) is taken as the MBC.

### 2.5 In vivo antimicrobial test of YG and sample collection

A pre‐experiment lasting for 1 month was carried out to confirm the safety of YG on *C. gibelio* when fed with a basal diet containing 800 mg/kg YG, and we found that the survival rate was 100%, no other symptoms were observed, along with its ingredients (Figure S1) and effects on growth performance of piglet [Qin et al., 2019]), we concluded that YG is safe to *C. gibelio* when fed with a basal diet containing 800 mg/kg YG.

Healthy *C. gibelio* were randomly allocated to four equal groups, including YG group, antibiotic group, negative control group and blank control group. Each group contained three replicates with 15 *C. gibelio,* respectively, and the trial lasted 14 days. All fish of YG group were fed with a basal diet containing 800 mg/kg (we referred to the addition in piglet, performed by Qin et al., 2019) YG (YG group), antibiotic group were fed with a basal diet containing 200 mg/kg Rifampicin serving as a positive control (PC group). The negative control group (NC group) were fed with basal diet. After 2 weeks, each fish in YG group, PC group and NC group was injected intramuscularly with 2 × LD50 viable *A. caviae.* Fish in blank control (BC group) was injected with equal volume of sterilized saline water. After 3 days post‐infection, the intestine of three individuals in each group was collected for microbial diversity analysis.

### 2.6 Microbial diversity analysis

Microbial DNA was extracted from intestine samples using the TIANamp Animals DNA Kit^™^ (Tiangen) according to the manufacturer's instructions. The final DNA concentration and purification were determined by NanoDrop 2000 UV‐vis spectrophotometer (Thermo Scientific), and DNA quality was checked by 1% agarose gel electrophoresis. The V3‐V4 hypervariable regions of the bacteria 16S rRNA gene were amplified with primers 338F (5′‐ACTCCTACGGGAGGCAGCAG‐3′) and 806R (5′‐GGACTACHVGGGTWTCTAAT‐3′) by thermocycler PCR system (GeneAmp 9700, ABI). The PCR reactions were conducted using the following program: 3 min of denaturation at 95°C, 27 cycles of 30 s at 95°C, 30 s for annealing at 55°C and 45 s for elongation at 72°C, and a final extension at 72°C for 10 min. PCR reactions were performed in triplicate 20‐μl mixture containing 4 μl of 5 × FastPfu Buffer, 2 μl of 2.5 mM dNTPs, 0.8 μl of each primer (5 μM), 0.4 μl of FastPfu Polymerase and 10 ng of template DNA. The resulted PCR products were extracted from a 2% agarose gel and further purified using the AxyPrep DNA Gel Extraction Kit (Axygen Biosciences) and quantified using QuantiFluor™‐ST (Promega) according to the manufacturer's protocol.

Purified amplicons were pooled in equimolar and paired‐end sequenced (2 × 300) on an Illumina MiSeq platform (Illumina) according to the standard protocols by Majorbio Bio‐Pharm Technology Co. Ltd. Operational taxonomic units (OTUs) were clustered with 97% similarity cut‐off using UPARSE and chimeric sequences were identified and removed using UCHIME (Edgar, 2010). Representative sequences defined by abundance from each OTU were aligned to the SILVA bacterial database using PyNAST (Caporaso, Bittinger, et al., 2010; Koetschan et al., 2014). The taxonomy of each 16S rRNA gene sequence was analysed by RDP Classifier algorithm against the Silva (SSU123) 16S rRNA database using confidence threshold of 70% (Caporaso, Kuczynski, et al., 2010). Shannon and Simpson indices were employed for diversity analyses, and the Ace and Chao1 indices were employed for richness analysis.

### 2.7 Statistical analysis

All data were sorted by excel 2016 after the initial order and analysed using SPSS 21.0 (2015, IBMSPSS Inc.). Variability in all the data was expressed as means ± SEM. Differences between mean values were compared using independent samples *T* test, and considered significant when *p* < .05.

## 3 RESULTS

### 3.1 Identification of *A. caviae* based on morphological, biochemical and 16S rRNA sequencing analysis

**Figure 1:**
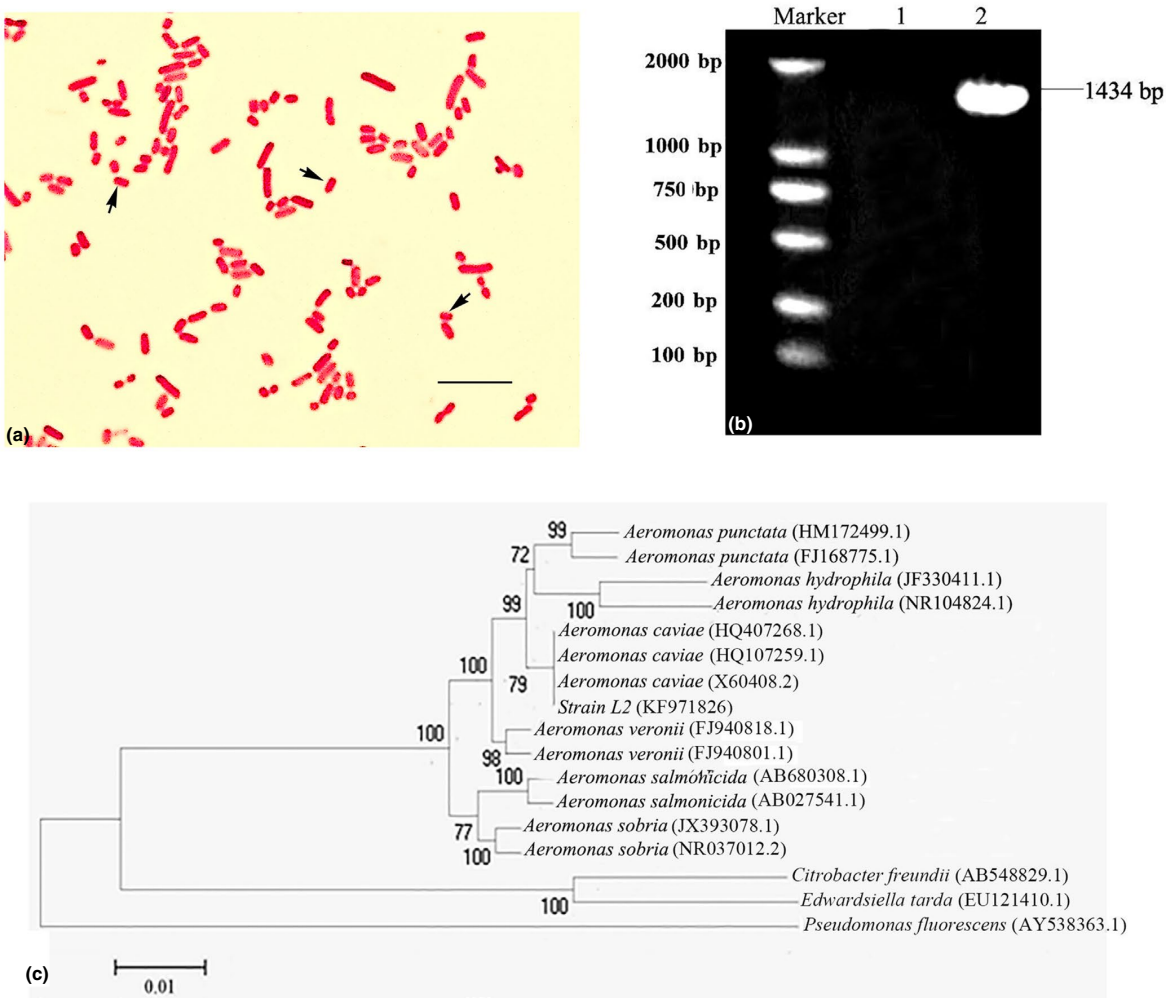
Identification of *Aeromonas caviae based on* Gram staining and 16S rRNA gene sequencing. (a) Gram staining of the strain L2. The black arrows showed rod‐shaped bacterium which were stained red, bar = 10 μm. (b) Agarose gel showing the amplification of 16S rRNA gene of strain L2. Lane M: DNA marker; Lane 1, negative control (without DNA template); Lane 2, 16S rRNA gene of strain L2. (c) Phylogenetic tree based on 16S rRNA sequences of strain L2 with other strains. Numbers in parentheses represent the sequences accession number in GenBank. The number at each branch points is the percentage supported by bootstrap. The scale bar represents 0. 01 nucleotide changes per position

Based on morphological, biochemical and 16S rRNA sequencing analysis, the three dominating bacterial colonies (L1, L2 and L3) were identified as *A. caviae* and concluded as clones of a single strain. Strain L2 was randomly selected to illustrate its morphological and biochemical characters of *A. caviae*. It had round and smooth circled shape on the agar plates, examination under microscope showed the bacteria were Gram negative (red) and rod shaped (Figure 1a). Based on physiological and biochemical identification, a total of 32 characteristics of strain L2 were highly consistent with those in *A. caviae* standard strain (Table 1), such as positive in oxidase and indol production, whereas negative in voges proskauer.

**Table 1:** Physiological and biochemical characteristics of strain L2

Test item	L2	A	Test item	L2	A	Test item	L2	A	Test item	L2	A
Oxidase	+	+	Citrate	−	−	Dulcitol	+	+	Voges Proskauer	−	−
Sorbose	−	−	Xylose	−	−	Lactose	−	−	Nitrate reduction	+	+
Sucrose	+	+	Sorbitol	−	−	KCN growth	+	+	Esculin hydrolysis	+	+
Mannitol	+	+	Melibiose	−	−	Trehalose	+	+	Lysine decarboxylase	−	−
1%NaCl	+	+	Rhamnose	−	−	Salicin	+	+	Arginine dihydrolase	+	+
0%NaCl	+	+	Maltose	+	+	D‐cellobiose	+	+	Ornithine decarboxylase	−	−
ONPG	+	+	Mannose	−	−	Arabinose	+	+	Gelatin hydrolysis	+	+
M‐R	+	+	Urease	+	+	Indol production	+	+	Gas production of glucose	−	−

Abbreviations: +, positive; −, negative; A, *Aeromonas caviae* standard strain; L2: strain L2.

The 16S rRNA gene of strain L2 was amplified by PCR and the products were shown in 1% agarose gel electrophoresis. According to the results of sequencing, the length of 16S rRNA gene was 1,434 bp (Lane 2, Figure 1b), no bands were detected in the negative control (Figure 1b, Lane 1). The sequence of 16S rRNA was uploaded to the NCBI GenBank (accession number KF971826), and submitted to BLAST for homology analysis and phylogenetic tree construction, 16S rRNA sequence of strain L2 has up to 99% similarity compared with *A. caviae*, and both of them are derived from the same cluster of phylogenetic tree (Figure 1c). But it is relatively distant from other *Aeromonas* bacteria, including *Aeromonas punctata* and *A. hydrophila*. All in all, the results provide further evidences that the strain L2 is *A. caviae*. By identifying the bacteria isolated from the liver of these experimentally infected *C. gibelio* characterized with bacterial septicaemia and hepatosplenomegaly, the pathogenic strain was identified as *A. caviae*.

### 3.2 Pathogenicity analysis of *A. caviae*

Cumulative mortality of *C. gibelio* in each group was recorded during 2 weeks of artificial infection, as shown in Table S1. Signs of disease *C. gibelio* were characterized as hepatosplenomegaly and abdominal hyperaemia. The results showed that 1 × 10^8^ CFU/ml was the minimum lethal concentration. When the concentration reached 1 × 10^7^ CFU/ml, the cumulative mortality was more than 50%. In the negative control group, no obvious signs or death were observed. According to Reed's method, the LD50 of *A. caviae* is 1.33 × 10^6^ CFU/ml. By identifying the bacteria isolated from these experimentally infected *C. gibelio*, the pathogenic strain was proven to be *A. caviae*.

**Figure 2:**
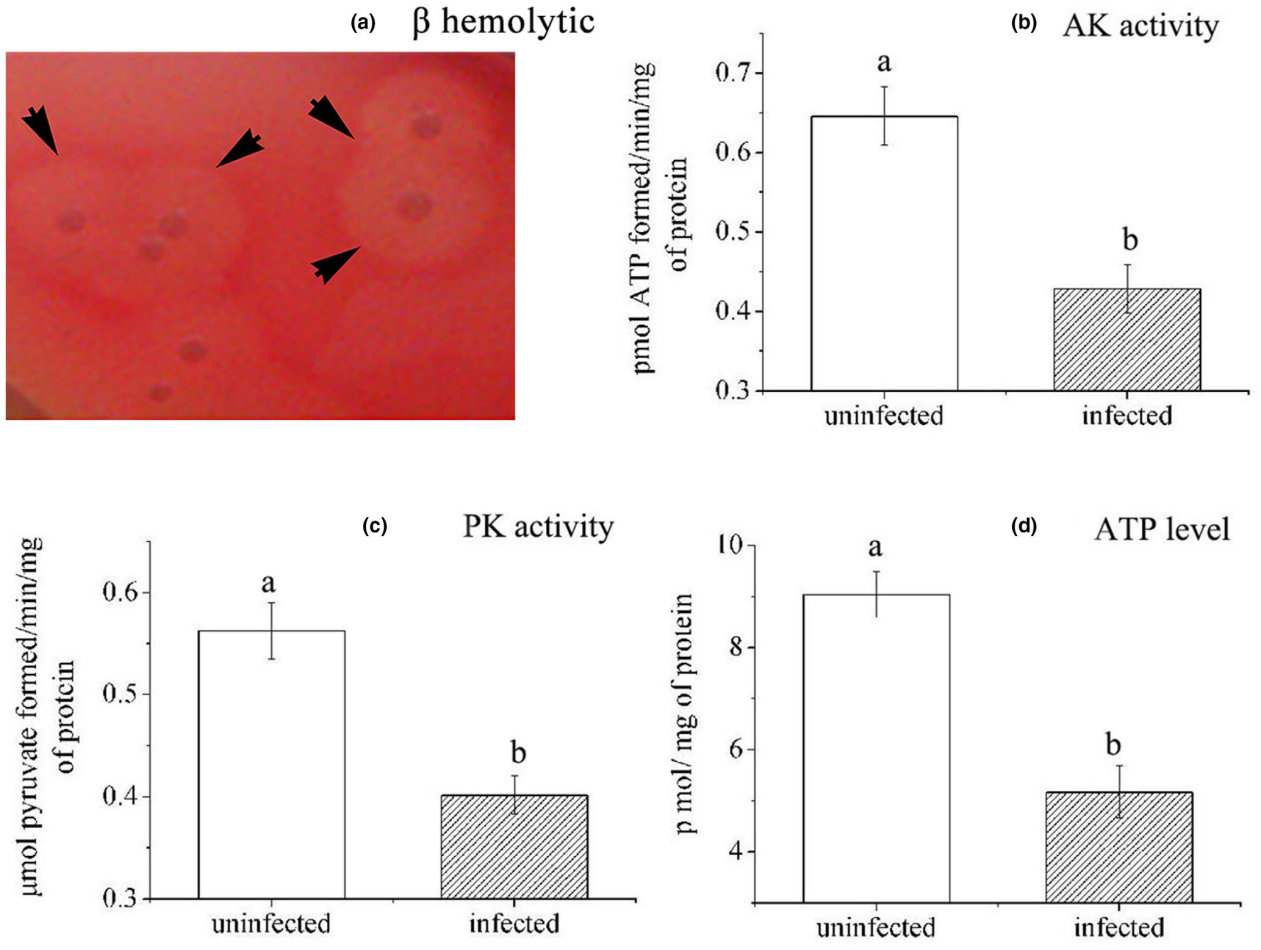
Pathogenicity analysis of *Aeromonas caviae*. (a) Haemolytic test of *A. caviae* ECPs. The black arrow showed β‐haemolytic circle on rabbit whole blood agar plate. (b–d) Hepatic AK, PK and ATP activities in liver of *Carassius auratus gibelio*. Error bars represented standard deviation (*SD*). Means with different letters were significantly different using two‐tailed Student's *t* test (*p* < .05). (b) Data represented pmol ATP formed/min/mg of protein (mean ± *SD*; *n* = 3). (c) Data represented and μmol pyruvate formed/min/mg of protein. (d) Data represented pmol/mg of protein. AK, adenylate kinase; ATP, adenosine triphosphate; ECP, extracellular product; PK, pyruvate kinase

After ECPs was inoculated in six different media at 28°C for 36 hr, transparent β‐haemolytic circles could be observed in 10% rabbit blood agar (Figure 2a), showing that ECPs of *A. caviae* had β‐haemolysis.

Liver of three randomly chosen diseased *C. gibelio* was sampled for analysis of AK and PK activities and ATP levels. The results showed that AK and PK activities significantly decreased (*p* < .05) by 33.7% and 28.6% in the liver of infected animals compared with liver in uninfected animals, respectively (Figure 2b,c). ATP levels in livers also showed an obvious decrease (42.8%, *p* < .05) post‐infection (Figure 2d).

### 3.3 In vitro antimicrobial activity of antibiotics and YG

**Figure 3:**
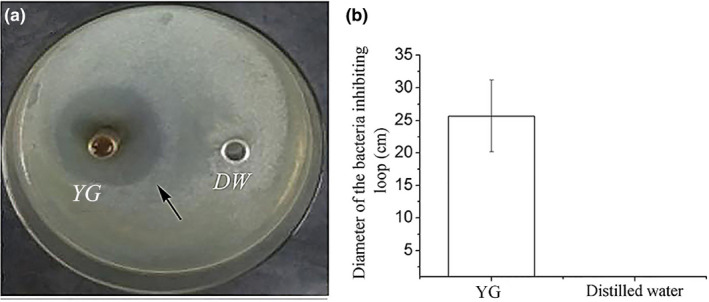
Antibacterial activity of YG in vitro was evaluated using Oxford cup method. (a) Determination of the inhibiting loop of YG. Black arrow indicated bacteria inhibiting loop. The negative controls were added equal volume of sterile DW. (b) Error bars represented *SD*. Data represented the diameter of inhibiting loop of YG (mean ± *SD*; *n* = 3). DW, distilled water; YG, yeast glycoprotein

A total of 30 different antibacterial drugs were used in the tests, the results showed that *A. caviae* was sensitive to seven drugs, including ceftazidime, cefotaxime, vancomycin, nitrofurantoin, aztreonam, rifampicin and amikacin (Table 2). After incubating at 28℃ for 24 hr, an obvious bacteria‐inhibiting zone appeared around Oxford cups adding YG in three culture dishes (Figure 3a). The assay was performed in triplicate, the average diameters of inhibiting zone was 25 mm (Figure 3b). No inhibiting zones were observed around negative controls adding DW.

**Table 2:** Sensitivity of *Aeromonas caviae* to antibiotics

Antibacterials	Dose, μg	Re, mm	Su, mm	IZS, mm	D	Antibacterials	Dose, μg	Re, mm	Su, mm	IZS, mm	D
Aztreonam	30	≤15	≥22	42	S	Minocycline	30	≤14	≥19	0	R
Sinomin	250	≤15	≥23	12	R	Cefoperazone	75	≤15	≥21	0	R
Claforan	30	≤14	≥23	36	S	Piperacillin sodium	100	≤17	≥21	0	R
Ampicillin	10	≤14	≥20	0	R	Chloramphenicol	30	≤12	≥18	0	R
Rifampicin	5	≤16	≥20	28	S	Sulfamethoxazole	3.73	≤24	≥24	0	R
Ofloxacin	5	≤12	≥16	0	R	Erythromycin	15	≤13	≥23	0	R
Cefalexin	30	≤14	≥18	0	R	Deoxycycline	30	≤12	≥16	0	R
Cefazolin	30	≤14	≥18	0	R	Clindamycin	2	≤14	≥21	0	R
Cefradine	30	≤14	≥18	0	R	Furazolidone	300	≤14	≥17	0	R
Amikacin	30	≤14	≥17	25	S	Neomycin	30	≤12	≥17	0	R
Kanamycin	30	≤13	≥18	0	R	Polymixin B	300	≤8	≥12	0	R
Cefuroxime	30	≤14	≥23	0	R	Norfloxacin	10	≤12	≥17	0	R
Macrodantin	300	≤14	≥17	29	S	Ceftazidime	30	≤14	≥18	41	S
Medemycin	30	≤13	≥18	0	R	Gentamycin	10	≤12	≥15	12	R
Tetracycline	30	≤14	≥19	0	R	Vancomycin	30	≤9	≥13	13	S

Abbreviations: D, resistance determination; IZS, inhibition zone size; R, resistance; Re, resistance; S, susceptibility; Su, sensitivity.

### 3.4 MIC and MBC of YG to *A. caviae*

**Figure 4:**
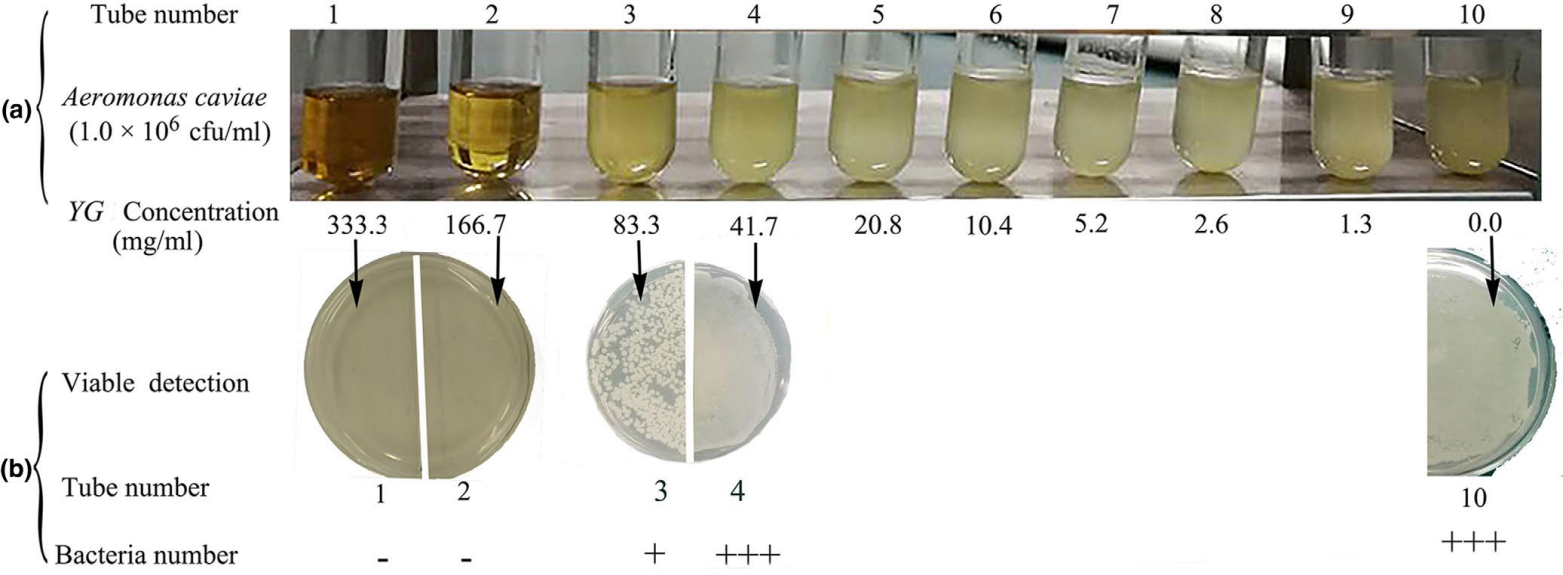
MIC and MBC of YG. (a) MIC of YG was determined by broth dilution methods. Tubes 1–9 was added serially dilutions of YG, respectively. Tube 10 without adding YG served as the positive control. (b) MBC detection. −: no bacteria; +: presence of bacterium; +++: actively growing bacterium. YG, yeast glycoprotein

After inoculation at 28°C for 24 hr, bacterial turbidity significantly decreased in tube 1–3 compared with tube 10 (positive control), and turbidity of tube 4–9 was similar to tube 10 (Figure 4a). To further determine the MBC of YG, the products in tube 1, 2, 3, 4 and 10 were sub‐cultured in LB agar plates at 28°C for 24 hr. The results showed no bacteria growth was detected in products from tube 1 and 2, some bacterial growth was observed in tube 3 and nearly equally actively growing bacterium with positive control plates (tube 10) was detected in products of tube 4 (Figure 4b). Therefore, the MIC and MBC of YG were 83.3 mg/ml and 166.7 mg/ml, respectively.

### 3.5 YG‐associated changes in *Aeromonas* in intestinal microbiota

**Figure 5:**
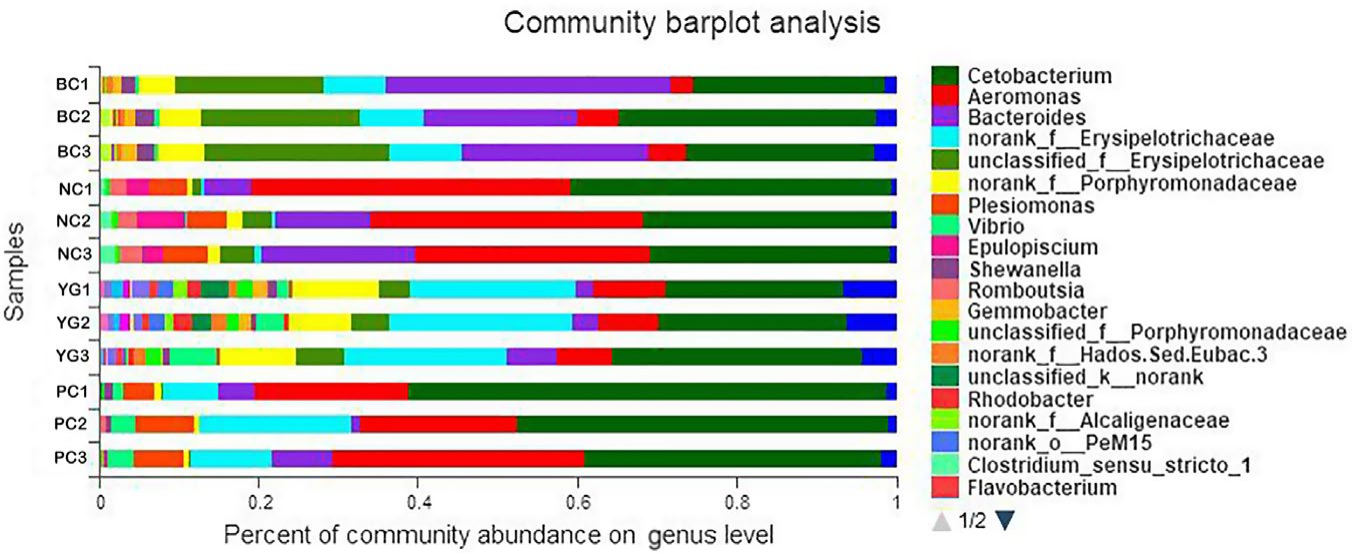
The influence of YG on the composition of intestinal microbiota at the genus level. BC: fish group feeding with the basal diet and injecting with sterilized saline water; NC, YG and PC group were injected with viable *Aeromonas caviae.* NC: artificial infected fish group feeding with the basal diet; YG: fish were fed with a basal diet containing 800 mg/kg YG. PC: fish were fed with a basal diet containing 200 mg/kg Rifampicin. Sequences that could not be classified into any known group were designated as “Unclassified”. Statistical comparisons were performed by the Mann–Whitney test. YG, yeast glycoprotein

The influence of YG on the intestinal microbiota at the genus level is shown in Figure 5. Community barplot analysis demonstrated *Cetobacterium* was the dominant bacteria of intestinal microbiota in all groups. *Aeromonas* became the dominant bacteria post–artificial infection (NC group) compared with BC group, and YG supplement (YG group) could more significantly decreased (*p* < .05) the relative abundance of *Aeromonas* than antibiotic group (PC group) did. Meanwhile, the microbial diversity and abundance of *Erysipelotrichaceae* and *Porphyromonadaceae* in YG group were highest among the four groups.

## 4 DISCUSSION

The bacterial colonies isolated from liver of diseased fish were identified as *A. caviae* according to colony morphology, biochemical and physiological characteristics*. A. caviae* was confirmed as a causative pathogen because satisfying Koch's postulates in normal *C. gibelio.* PCR was performed to identify the bacterial isolate at molecular level using primers specific to 16S rRNA gene of *A. caviae.* To our knowledge, this is the first pathogenic diagnosis of *A. caviae* in *C. gibelio*, and there is limited research on ecological prevention and control of *A. caviae* in aquaculture even though it occurs ubiquitously in aquaculture industry (Ringø & Vadstein, 1998).

Reproduction of infection demonstrated that normal *C. gibelio* was susceptible to *A. caviae* infection, the LD50 of *A. caviae* for *C. gibelio* is 1.33 × 10^6^ CFU/ml, indicating that *A. caviae* was highly virulent to *C. gibelio*. The production of haemolytic toxins has been regarded as indication of pathogenic potential, and all *Aeromonas* isolates with haemolysin‐positive genotype were virulent in the suckling mouse assay model (Wong et al., 1996). In the present pathogenicity analysis, haemolytic test of ECPs showed that *A. caviae* possessed β‐haemolysis, which was potentially associated with the signs of infected *A. caviae*, such as haemorrhage in the liver. AK and PK are two key enzymes linked to the communication between sites of ATP generation and ATP utilization, and a recent study found that experimental infection by *A. caviae* could decrease AK and PK activities in experimentally infected silver catfish (Baldissera et al., 2018). In this study, we observed that hepatic AK and PK activities in *C. gibelio* were inhibited by *A. caviae*, which resulted in decreased availability of hepatic ATP. Therefore, the inhibition of hepatic AK and PK activities by *A. caviae* infection might contribute to disease pathogenesis in *C. gibelio*.

The antibiotic sensitivity test showed that *A. caviae* was multi‐antibiotic, and its drug‐resistant characteristics were different from *A. caviae* isolated from other species (Huang, Wang, Zheng, & Huang, 2010; Yu & Ma, 1997). As *A. caviae* infecting *C. gibelio* has multiple antibiotic resistance, development of a microecological preparation is an alternative and promising prophylactic method to provide protection against *A. caviae*. A previous study showed that YG supplement could obviously increase the relative abundance of *Lactobacillus* in weaned piglets (Qin et al., 2019), it was not clear about its effect on pathogen and intestinal microbiota of aquatic animals. This study showed that YG could effectively inhibit *A. caviae* replication in vitro. Community barplot analysis demonstrated that YG supplement could more significantly increase the microbial diversity in the gut and decrease the relative abundance of *Aeromonas* than antibiotic did in vivo. Interestingly, we found that bacterial abundance of *Erysipelotrichaceae* and *Porphyromonadaceae* was highest in the YG supplement group. According to the previous studies, these two key bacterial families belong to indigenous microbiota and are related to4 the digestion of protein and energy (Bermingham, Maclean, Thomas, Cave, & Young, 2017; Sakamoto, 2014). Therefore*,* YG supplement could efficiently inhibit *A. caviae* both in vivo and in vitro, and contribute to intestine health. Further study concerning optimizing the adding level of YG supplement is needed.

To conclude, this is the first report that *C. gibelio* is susceptible to *A. caviae* infection. Its β‐haemolysis and decrease of AK and PK activities might be potentially associated with virulence and disease pathogenesis. *Aeromonas caviae* isolated from *C. gibelio* was a type of multiple antibiotic‐resistant bacteria strain and highly virulent to *C. gibelio.* Fortunately, it could be effectively inhibited by YG in vivo and in vitro, providing a potentially safe and effective alternative method to traditional antibiotics. These conclusions shed light on ecological control and pathogenesis of *A. caviae* in aquaculture.

## CONFLICT OF INTEREST

The authors declare no conflicts of interest.

## AUTHORS’ CONTRIBUTIONS

Yun Li, corresponding author, involved in the study design and concept, statistical analysis and manuscript authorship. Ronghua Wu and Junyu Shen involved in the project implementation, data acquisition, editing and major revisions of manuscript. Dandan Tianand Jiaqian Yu involved in the fish handling and data sorting. Tao He and Jianhua Yi involved in analysis of report and technical support. All authors have read and approved the final version of this manuscript.

## ETHICAL APPROVAL

This study was conducted in strict accordance with the recommendations in the Guide for the Use of Experimental Animals of Southwest University, China. Before sacrificing and handling, experimental *C. gibelio* were anesthetized with ethyl 3‐aminobenzoate methanesulfonic acid (MS222, Sigma, USA), and all efforts were made to minimize suffering.

## Supporting information

Table

Figure
