# Supplementary material for: A potential alternative to traditional antibiotics in aquaculture: Yeast glycoprotein exhibits antimicrobial effect in vivo and in vitro on Aeromonas caviae isolated from Carassius auratus gibelio
Source: Vet Med Sci. 2020 Mar 24;6(3):639–48. doi: 10.1002/vms3.253 (PMC7397907; doi:10.1002/vms3.253)
Supplement: Supplementary file 1 — Table [file VMS3-6-639-s001.doc]

**Table S1. Results of artificial infection of *A. caviae* in *C. gibelio***

| Group | Concentration( cfu /mL) | Number of ***C. gibelio*** | Death number | Mortality(%) |
| --- | --- | --- | --- | --- |
| 1 | 1 × 10^9^ | 20 | 20 | 100 |
| 2 | 1 × 10^8^ | 20 | 20 | 100 |
| 3 | 1 × 10^7^ | 20 | 17 | 85 |
| 4 | 1 × 10^6^ | 20 | 9 | 45 |
| 5 | 1 × 10^5^ | 20 | 4 | 20 |
| 6 | PBS | 20 | 0 | 0 |
