# Supplementary figures and images for: A potential alternative to traditional antibiotics in aquaculture: Yeast glycoprotein exhibits antimicrobial effect in vivo and in vitro on Aeromonas caviae isolated from Carassius auratus gibelio
Source: Vet Med Sci. 2020 Mar 24;6(3):639–48. doi: 10.1002/vms3.253 (PMC7397907; doi:10.1002/vms3.253)

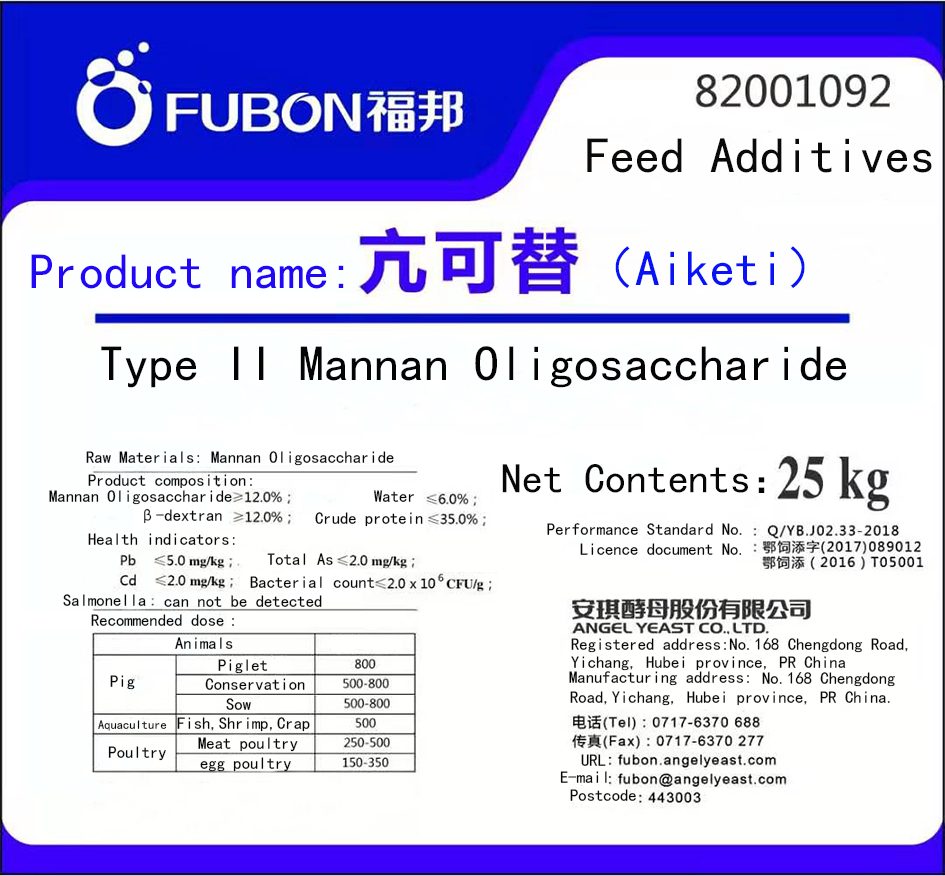

Supplement: Supplementary file 2 — Figure [file VMS3-6-639-s002.tif]
